# P-1896. Comparing Clinical Diagnoses of Intraamniotic Infections to the Guideline Definitions

**DOI:** 10.1093/ofid/ofae631.2057

**Published:** 2025-01-29

**Authors:** Grace Pazienza, Lance Schacht, Mattie Jo Thomas, Jennifer Palomo, Sean Stuart, Sarah Withers, Amy H Crockett, Pamela Bailey

**Affiliations:** Prisma Health - University of South Carolina SOM - -, West Columbia, South Carolina; University of South Carolina School of Medicine, Columbia, South Carolina; University of South Carolina SOM, Columbia, South Carolina; Prisma Health - University of South Carolina SOM - -, West Columbia, South Carolina; Prisma Health - University of South Carolina SOM - -, West Columbia, South Carolina; Prisma Health, Greenville, South Carolina; Prisma Health - University of South Carolina SOM, Greenville, South Carolina; Prisma Health Richland - University of South Carolina, Columbia, South Carolina

## Abstract

**Background:**

Intraamniotic infection (IAI) or chorioamnionitis is an intrapartum clinical diagnosis. Treatment delay for confirmatory test results can lead to maternal and fetal morbidity. The American College of Gynecology (ACOG) defines “suspected IAI” as persistently elevated temperatures plus findings of purulent cervical discharge, maternal leukocytosis, fundal tenderness, and maternal or fetal tachycardia. These variable and relatively complex diagnostic criteria may introduce clinical confusion and lead to overtreatment. We sought to evaluate compliance with ACOG guidelines in our hospital.

**Table 1:**
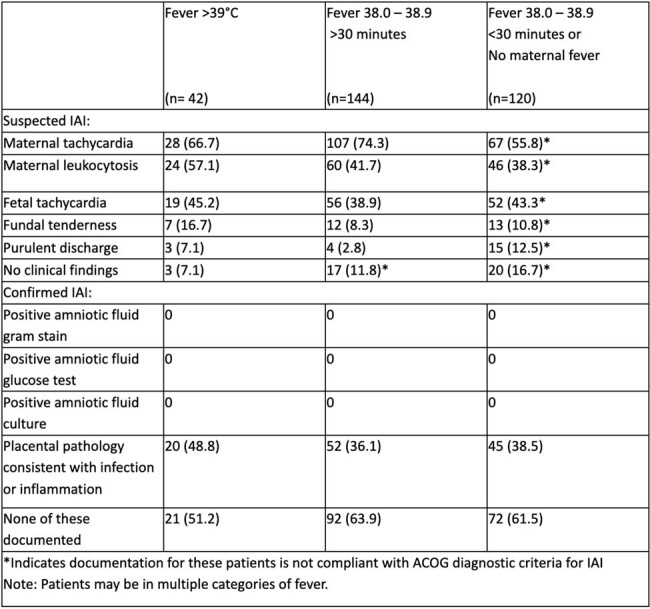
Diagnostic criteria for IAI including categories of maternal fever and clinical findings

**Methods:**

A retrospective cohort study was planned. Patients with IAI were identified by ICD diagnoses, and screened for appropriateness. Manual chart abstraction was performed to identify diagnostic criteria for IAI per ACOG guidelines. Additionally, culture and pathology results were reviewed when available. Comparisons between groups were made using chi square.

**Results:**

301 patients with IAI were identified between Feb 2016 and Oct 2023. Most (n=281, 93.4%) met ACOG diagnostic criteria but 20 (6.6%) did not have documented indications for initiation of treatment. There is no statistical significance between the number of symptoms and fevers (p=0.55) between groups (Table 1).

Few (n=59, 19.6%%) patients had cultures collected, and of these 35.5% (n=21) were positive including placenta (n=10, 47.6%) and blood (n=2, 9.5%). There was not a difference in patients with positive cultures compared to patients with negative or no cultures in fevers (p=0.77) or number of suspected IAI signs (p=0.11).

**Conclusion:**

The diagnostic criteria for IAI should be refined or reevaluated, particularly in light of their complexity in accurately diagnosing IAI. This is especially important in light of antimicrobial stewardship initiatives in an effort to improve patient care, as well as an introduction of rapid diagnostics improving earlier identification of organisms in blood cultures which could significantly impact organism identification earlier than traditional microbiology.

**Disclosures:**

All Authors: No reported disclosures

